# Finding overlapping communities in multilayer networks

**DOI:** 10.1371/journal.pone.0188747

**Published:** 2018-04-25

**Authors:** Weiyi Liu, Toyotaro Suzumura, Hongyu Ji, Guangmin Hu

**Affiliations:** 1 University of Electronic Science and Technology of China, School of Communication & Information Engineering, Chengdu, Si Chuan, China; 2 IBM Thomas J. Watson Research Center, Network Science and Big Data Analytics Department, New York, United States of America; University of Bristol, UNITED KINGDOM

## Abstract

Finding communities in multilayer networks is a vital step in understanding the structure and dynamics of these layers, where each layer represents a particular type of relationship between nodes in the natural world. However, most community discovery methods for multilayer networks may ignore the interplay between layers or the unique topological structure in a layer. Moreover, most of them can only detect non-overlapping communities. In this paper, we propose a new community discovery method for multilayer networks, which leverages the interplay between layers and the unique topology in a layer to reveal overlapping communities. Through a comprehensive analysis of edge behaviors within and across layers, we first calculate the similarities for edges from the same layer and the cross layers. Then, by leveraging these similarities, we can construct a dendrogram for the multilayer networks that takes both the unique topological structure and the important interplay into consideration. Finally, by introducing a new community density metric for multilayer networks, we can cut the dendrogram to get the overlapping communities for these layers. By applying our method on both synthetic and real-world datasets, we demonstrate that our method has an accurate performance in discovering overlapping communities in multilayer networks.

## Introduction

**Fig 1:**
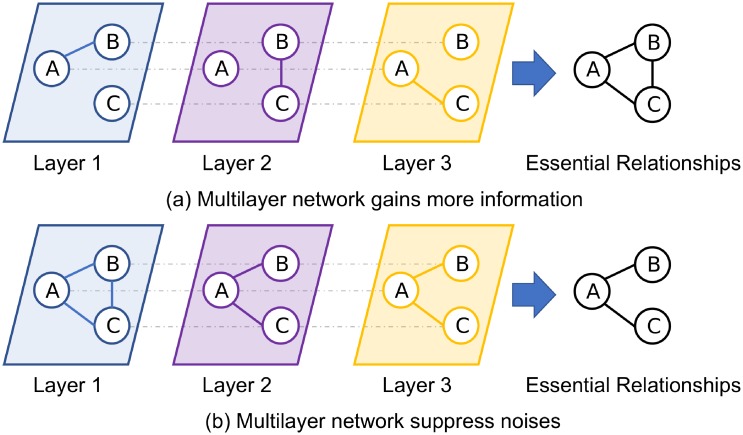
A toy example to show two advantages of multilayer network analysis. Fig (a) shows that by co-analysis the layers, node A, B, C are within the same community. Fig (b) shows that the edge (B, C) in layer 1 is the noise edge.

In the past 20 years, complex network analysis has become a vital tool for investigating complex systems in the social and natural world [1–3]. Meanwhile, discovering communities in these systems has become a primary and prior object to help us understand how network structure relates to system behaviors [4, 5]. With the deepening of the research, more and more researchers have come to realize that simply uncovering communities in a single network is insufficient to analyze the structures and system behaviors in the real world [6–12]. Hence, community discovery methods for multilayer networks have been introduced to leverage various relationships to get more accurate results [11, 13]. Here, multilayer networks represent a set of networks composed of a collection of nodes and various types of relationships between these nodes. Furthermore, each layer stands for a particular type of relationship between these nodes [12, 13]. However, the definition of communities for multilayer networks still depends on the problem we intend to solve. Generally speaking, from a topological structure standpoint, a community is a subset of nodes that have more inner edges [5, 14, 15]; from an information theoretic view, however, by considering the probability flow of random walks on a network as a proxy for information flows in a real system, we can define a community in the system as a module that can constrain the flow of information for a relatively long time [16–18]. As multilayer network contains more (rich) topological information among the nodes, finding communities within it is also important. That is, each layer represents a typical relationship among the set of nodes. Please pay attention that for multilayer network, it is not strictly needs that each layer of the network has a same set of node, by co-analyzing these layers, one can not only access to more aspects (relationships) among the set of node, but also has the opportunity to discover essential relationships among nodes. In addition, as different layers can suppress the random noises embed in a single layer, multilayer network analysis enhances signal-noise separation during the analysis process [12]. Take Fig 1 as an example, here we use two sub-figures to demonstrate the two advantages to do multilayer network analysis. For each sub-figure, we use three layers to show the different relationships among node set {*A*, *B*, *C*}, along with the essential relationship among the node set. Consider Fig 1(a), each layer represents two of three nodes are connected, while the other one an isolated node. If we only analysis one layer, it is easily to ignore the relationship between the connected and unconnected nodes, which is hard to extract the essential relationships among these nodes. Consider Fig 1(b), it is clearly to see that node-pair (*A*, *B*) and node-pair (*A*, *C*) are frequently connected, while node-pair (*B*, *C*) is not that frequent. Hence, the essential relationship is a tangle *BAC*. However, if applied the community detection algorithm only on layer 1, it is hard to discover such essential relationships.

Nowadays, community discovery methods for multilayer networks involve two stages, namely, “aggregation analysis” and “network co-analysis” (see Refs. [12, 13] for additional details). In the aggregation analysis stage, researchers often use “network aggregation” [19–22] or “community aggregation” [23, 24] to abstract communities. The former aggregates all networks into a single (weighted) network and then applies existing community discovery methods to reveal the communities in multilayer networks. The latter finds communities in each layer, and then merges these communities together to discover the communities in multilayer networks. However, aggregating networks may weaken or even result in the loss of the structural topology information in each layer [23]. Moreover, merging communities in each layer does not consider the fact that the behaviors of nodes may vary from one layer to another. Further, both stages may ignore the interplay between different layers [18]. To address the problem in the aggregation process, more and more researchers have begun to use network co-analysis to directly discover communities in multilayer networks. The representative algorithms include “bridge detection” [25], “tensor decomposition” [26–28], “multi-slice modularity” [11, 29–32], etc. Nevertheless, despite the fact that these co-analyzing algorithms can provide better results than aggregation, they may contain some flaws internally. Because bridge detection and tensor decomposition must use the number of communities as the prior knowledge, multi-slice modularity can only detect non-overlapping communities. On the other hand, as real networks always have pervasive overlapping phenomena [33–37], community discovery methods for a single network have already been well studied (for a review, see Refs. [35]). For multilayer networks, however, the use of network co-analysis to reveal overlapping communities has just started. In 2015, Domenico et al. extended the Infomap method to multilayer networks to find overlapping communities in networks [18]. In 2016, Magnani et al. extended clique percolation to multiplex networks [37].

In addition, the structural analysis or evolutionary dynamics including epidemic spread, synchronization or game cooperation have made some great advances by using the power of community detection on multilayer networks. For example, regarding the collective game cooperation on multilayer networks, Wang et al. [38] conducted an experiments on analyzing the evolution of cooperation in the spatial public goods game on interdependent lattices on multilayer networks, and Xia et al [39] used multilayer networks to depict the suitable coupling between networks.

In this paper, we propose a new community discovery method for multilayer networks, which not only forms the hierarchical structure of multilayer networks but also can find potential overlapping communities in these networks. It is worth noting that, in a single network, an edge is used to clearly represent the connection relation that two nodes (aka “node pair”) share. However, with the increase in the number of network layers, the connection relation between two nodes is no longer a binary problem anymore, but now faces three situations: 1) a node pair shares no edge in all layers; 2) there exists an edge between the node pairs in each layer; and 3) there are several edges that a node pair shares only in several layers. Generally, in multilayer networks, node pairs have too many situations to deal with. To avoid this problem, we propose the use of “edges” in multilayer networks instead because regardless of how node pairs may vary in different layers, an edge always represents a simple “exist-or-not” binary idea. We conjecture that two edges that do not share a common node carry no structure information; therefore, herein, we only take an “edge pair” into consideration. An edge pair represents two edges that have a common node, and it is worth noting that this edge pair can exist in the same layer or in cross layers.

Our approach can be divided into three main steps:

1. Extraction of all edge pairs in multilayer networks: here, an edge pair can exist in the same layer (two edges share a common node) or in different layers (different layers have two nodes with a common name node).
2. Calculation of the similarity of all edge pairs: since an edge pair may exist both in the same layer and in cross layers, it is necessary to consider all the situations that occur in multilayer networks when calculating the similarity.
3. Formation of hierarchical structures and search for overlapping communities: by using the edge pair as the fundamental elements, we can form the hierarchical structure for multilayer networks by these edge-pair similarities. Then, by extending the community density in a single network [35], we can present the community density for a multilayer network. Finally, we can find overlapping communities by maximizing this density in the hierarchical structure.

By using both synthetic and real-world datasets to evaluate our method, we can demonstrate that our method can produce accurate results in revealing overlapping communities in multilayer networks. In general, this method has four contributions:

- There is no need to prescribe (or know) the number of communities in the networks.
- This method can leverage the unique topological structure information in each layer.
- This method is the first to quantify the “community density” in multilayer networks.
- This method can detect overlapping communities in multilayer networks.

## Materials and methods

In this section, we first elaborate the definition of a community in multilayer networks and represent a description of “what a link-pair is” in multilayer networks; then, we give a quick overview of two popular community detection methods for multilayer networks. Finally, by introducing “link-pair calculation” and “community evaluation,” we can elaborate the details of each step of our algorithm and analyze the time complexity of our method.

### Community definition and link-pair definition

In this section, we first elaborate the definition of a community in multilayer networks, and then we give the definition of link pairs.

#### Community definition

The question “what is a community in multilayer networks?” is a sophisticated one: Each layer can have its communities, and these communities may vary from one layer to another. As the communities’ behaviors differ from each other from different points of views, in our opinion, the definition of a community in networks should depend on the particular task. In general, there are two definitions: one is that the community should be a fixed one across all layers, and the other is that the community can vary from one layer to another, where different layers can have their own communities. Here, the former definition mainly stresses the same behaviors for nodes in all networks. Moreover, there exist only tiny differences for these nodes in different layers. On the contrary, the latter definition mainly stresses the difference for these nodes in cross layers. In our opinion, we believe that multilayer networks are only a reflection of a single relationship that is shared by a group of nodes. For example, consider the relationships between the functions “Follow,” “Retweet,” and “Comment” from the online social network, Twitter. In our opinion, although we take three different relationships into consideration, these three–layer multilayer networks are only a reflection of the “friendship” between nodes. Therefore, in this paper, we treat communities as fixed communities.

#### Link-pair definition

**Fig 2:**
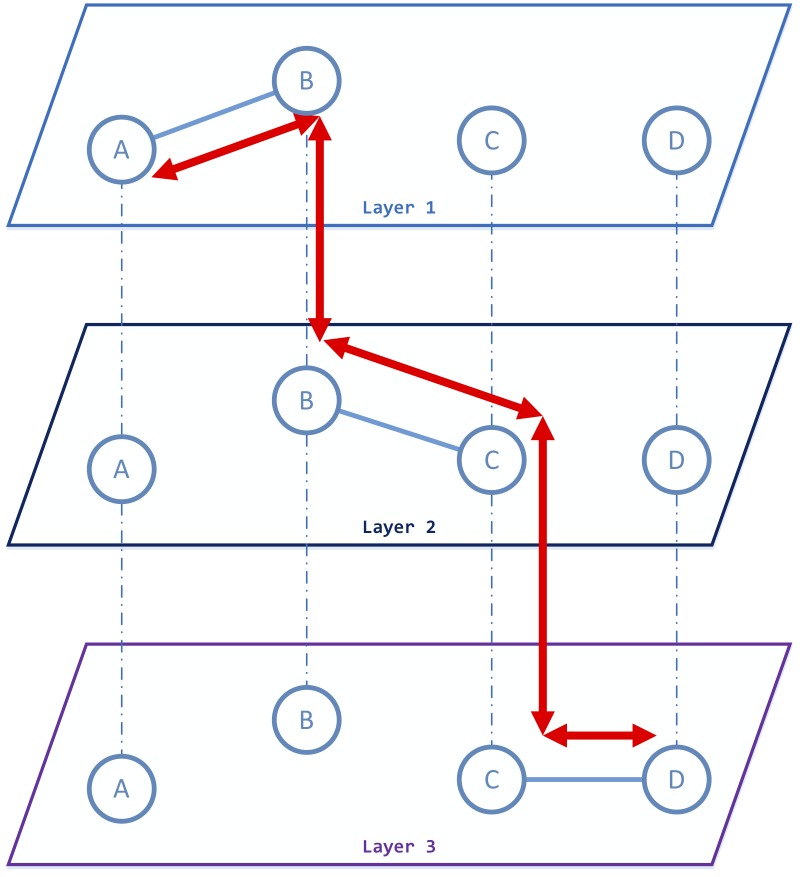
A toy example of a node pair that is reachable in multilayer networks: the information can start with node *A* and finally reach node *D*. By using the same name node *B* in layer 2, the information can reach node *C* in layer 2. And then, using the same node *C* in layer 3, this information will reach node *D* in layer 3 at last.

In general, “node pairs” and “edge pairs” are also more complicated in multilayer networks. For example, for node pairs, it is obvious that there is typically more than one type of edges for a node pair in multilayer networks, which means that the existence of an edge is no longer a binary question, but has become a multi-valued one. At the same time, owing to multilayer characteristics, we conjecture that the nodes in a node pair can also communicate with each other even if there exists no direct edge within this node pair in each layer. Take Fig 2 as an example. Although there exists no direct link from node *A* to node *D* within any layers, an information flow path (indicated in red) exists from node *A* to node *D* via the three-layer network, which implies that nodes *A* and *D* do have a way to communicate across layers.

The primary reason for this case is that, in multilayer networks, we can form edge pairs not only from the “same layers” but also from the “cross layers.” Here, we define “link pairs” instead of “edge pairs” to depict the connection behaviors for two edges in multilayer networks: A link pair is defined as a combination of “same-layer edge pairs” and “cross-layer edge pairs” from multilayer networks. In our opinion, leveraging link pairs in multilayer networks allows us to preserve the unique topological structure in each layer and to take the interplay between layers into consideration at the same time. As shown in Fig 2, by using the link pairs in networks, we can detect two cases of interplay from node *A* to node *D* on the basis of three layers. However, such interplay does not exist in any of the layers.

### Comparison of methods

To evaluate our method, we compared it to two different types of well-known community discovery methods: one is the “multi-slice modularity”-based Louvain method from network co-analysis. This method attempts to find communities by leveraging all structure information across layers. The other is link-community discovery from network aggregation. This method finds communities in an aggregate weighted network.

#### The “Multi-slice Modularity”-based Louvain method

Mucha et al. presented a multi-slice generalization of modularity [11] to study the community structure in multilayer networks (see Eq 1). Here, *i* and *j* represent all nodes, and *s* and *r* range over all layers. *C*_*jsr*_ is a binary value {0, *ω*} indicating the absence (0) or presence (*ω*) of interconnection between layers. *K*_*is*_(*K*_*js*_) is the degree of node *i*(*j*) in layer *s*, *m*_*s*_ is the number of edges in layer *s*, *γ*_*s*_ is a resolution parameter, and *μ* is a normalization factor.

$$\begin{array}{c} Q_{multi-slice}=\frac{1}{2\mu }\left\{\left(A_{ijs}-\gamma_{s}\frac{K_{is}K_{js}}{2m_{s}}\right)\delta_{sr}+\delta_{ij}C_{ij}\right\}\delta \left(g_{is},g_{jr}\right) \end{array}$$ (1)

The exact multi-slice modularity optimization is an NP-complete problem. To address this, we upgrade the Louvain (or BGLL) method to find communities in multilayer networks. For a multilayer network, by initially considering each node as a community and regarding the same-name node in different layers as two distinct nodes, we can implement this algorithm using just two steps repeated iteratively. Here, “same-name” node means that the node exists in two different layers.

**step 1** Find the local maximum of *Q*_*multi*−*slice*_ for the current multilayer networks.
  1. For each node *i* in layer *s*, we calculate the maximum local *Q* by moving node *i* to its neighbor *j*’s community. Moreover, we treat same-name node *i* in different layers as node *i*’s neighbors too.
  2. This step is performed iteratively until a local maximum of *Q* is reached.
**step 2** Deduct nodes and calculate their weight for the current multilayer networks.
  1. For each layer, we reduce the nodes in the same community into one node.
  2. For edges in the same layer, we use the sum of the weights of all the edges between communities corresponding to the new nodes *i* and *j* to represent the weight of the new edge.
  3. For a self-loop edge in the new node, we sum all the edges’ weights and all the nodes’ self-loop weights within the corresponding community to obtain the weight of the new self-loop edge.
  4. For the edges of the cross layers, the weight of this inter-layer edge between node *i* in layer *s* and node *j* in layer *r* is given by the sum of the weights of the edges between communities corresponding to node *i* and node *j*.

#### Link community discovery in a unified graph

By merging all layers into a weighted network, we can upgrade the link community detection method proposed by Ahn et al. [35] to find overlapping communities in it. Here, the “weight” of an edge represents the number of edges for a node pair in all layers.

Given a weighted graph *G*, Eq 2 shows how to calculate the similarity of an edge pair {*e*_*ik*_, *e*_*kj*_} in the weighted graph. Here, *n*_+_(*i*)/*n*_+_(*j*) stands for the group of nodes that contains node *i*’s/*j*’s neighbors and itself. *w*(*e*_*im*_) and *w*(*e*_*mj*_) stand for the weight of the edges *e*_*im*_ and *e*_*mj*_, respectively.

$$\begin{array}{c} weighted\_S\left(e_{ik},e_{kj}\right)=\frac{\sum_{m\in \{n_{+}\left(i\right)\cap n_{+}\left(j\right)\}}\left[w\left(e_{im}\right)+w\left(e_{mj}\right)\right]}{\sum_{m\in \{n_{+}\left(i\right)\cup n_{+}\left(j\right)\}}\left[w\left(e_{im}\right)+w\left(e_{mj}\right)\right]} \end{array}$$ (2)

On the basis of this similarity, we use single-linkage hierarchical clustering to find communities in this weighted graph. By initially assigning each link to its own community, we can merge the pair of edges with the largest similarity. Then, by leveraging a dendrogram to store this clustering process, we can construct the hierarchical structure for the graph. Finally, by maximizing the community density in Eq 3 for each height in a dendrogram, we can extract the overlapping communities for this weighted network. Here, *n*_*c*_ and *e*_*c*_ represent the node set and edge set, respectively, for this community (*C*).

Density(C)=|ec|-(nc-1)(nc2)-(nc-1) (3)

### Our method

In this section, we first introduce the two core contributions of our community detection method: “link-pair calculation” and “community evaluation.” Then, we describe our algorithm.

#### Link-pair calculation

There are three steps for calculating the similarities of link pairs: 1) Merge all layers into a unified network; 2) extract all link pairs; and then 3) calculate the similarities for link pairs.

For the merging part, we use Eq 4 to determine if there is an edge between two nodes. Here, {*a*_*ij*_} stands for the adjacency matrix for the unified network and $\left\{a_{ij}^{l}\right\}$ stands for the adjacency matrix for layer *l*. This equation determines if there exists an edge in any of a layer, and if one exists, we should add the edge to the unified graph. The advantage here is that we can easily extract all link pairs in multilayer networks, as these link pairs have already been turned into edge pairs in this unified graph by Eq 4.

$$\begin{array}{c} a_{ij}=\{\begin{array}{cc} & 1,\forall l\in L,\exists a_{ij}^{l}=1\\ & 0,else \end{array} \end{array}$$ (4)

**Fig 3:**
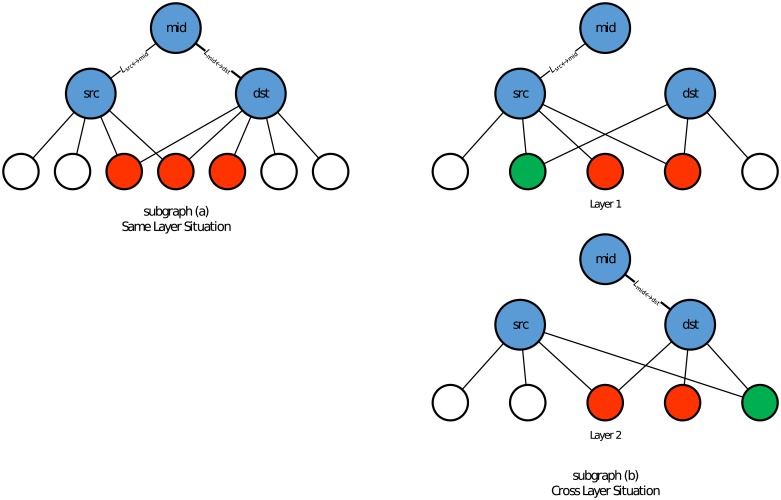
A toy example of common neighbors in a link pair.

For the similarity calculation part, as the similarity of same-layer edge pairs and cross-layer edge pairs is probably different from each other, we calculate their similarities separately. Take Fig 3 as an example. This toy example shows same-layer and cross-layer situations for a link pair {*L*_*src*↔*mid*_, *L*_*mid*↔*dst*_}, which is formed by three nodes {*src*, *mid*, *dst*}. For each sub-graph, we use red nodes to demonstrate the common neighbors of nodes *src* and *dst* whose structure information needs to be leveraged in the similarity calculation, whereas the white and green nodes demonstrate the other neighbors of *src* and *dst*. It is natural to see that, in sub-graph (a), all three common neighbors for *src* and *dst* should be used in the similarity calculation. In sub-graph (b), however, the green nodes represent the common neighbors only in one layer, whereas the red nodes represent the common neighbors in both layers. Here, we only calculate the similarities for the red nodes.

Here, we use the *Jaccardsimilarity* to calculate the similarity of the link pair {*L*_*src*↔*mid*_, *L*_*mid*↔*dst*_} (see Eq 5). In addition, we introduce a moderator parameter *α* ∈ [0, 1] to weigh the cross-layer similarity *crosslayerSim*(*L*_*src*↔*mid*_, *L*_*mid*↔*dst*_).

$$\begin{array}{c} SIM\left(L_{src↔mid},L_{mid↔dst}\right)=samelayerSim\left(L_{src↔mid},L_{mid↔dst}\right)\\ +\alpha \times crosslayerSim(L_{src↔mid},L_{mid↔dst}) \end{array}$$ (5)

#### Community evaluation

Although “(multi-slice) modularity” is quite useful and popular for community evaluation, this metric only focuses on nodes. As our method focuses on link pairs, in our opinion, the “modularity” may not be sufficient enough to depict the communities for our case. This, in fact, is the very reason why we want to propose our own evaluation method for communities in multilayer networks. An alternative approach is to use the community density proposed by Ahn [35] for each layer and then sum them up to get the community density for multilayer networks. However, in our opinion, the density of a community should reflect the tightness of connections between the nodes in all layers, whereas a simple “summing up” may not able to achieve this goal as it may suffer from a number of statistical problems; for example, a large community density that has been “summed up” cannot distinguish whether such community is comparatively dense in all layers or is just denser in some layer(s).

In this paper, we propose a new community density metric for multilayer networks: Given a community in multilayer networks *C* = {*C*^1^, *C*^2^, ⋯, *C*^*l*^}, where $C^{l}=\left(V_{C}^{l},E_{C}^{l}\right)$ stands for the node number $V_{C}^{l}$ and edge number $E_{C}^{l}$ of this community (*C*) in layer *l*. Moreover, it is natural to deduce that the minimum edge number of this community (*C*^*l*^) is $\text{min}\left(E_{C}^{l}\right)=V_{C}^{l}-1$ and that the maximum edge number of *C*^*l*^ is max(ECl)=(2VCl). A basic idea for evaluating the community density is to calculate the connection possibility between the $V_{C}^{l}$ nodes and to use the minimum and maximum numbers of links in community *C*^*l*^ to normalize such possibility. Here, we use Eq 6 to depict the community density. Note that, as all layers must be taken into consideration simultaneously, each value in these fractions should contain all the layers’ information in the first place. In the rest of our method, we mainly use this metric to evaluate the outcomes for our method. From this Eq 6, we can see that the denser a community in multilayer networks is, the larger the outcome of this equation will be.

$$\begin{array}{c} Density\left(C\right)=\frac{\sum_{l=1}^{L}E_{g_{l}}-\sum_{l=1}^{L}\text{min}\left(E_{g_{l}}\right)}{\sum_{l=1}^{L}\text{max}\left(E_{g_{l}}\right)-\sum_{l=1}^{L}\text{min}\left(E_{g_{l}}\right)} \end{array}$$ (6)

#### Detailed algorithm

There are three major steps in our method. The core steps are the “link-pair similarity calculation” and “community evaluation” described above. The third one is “hierarchical structure construction.” In general, by calculating all link-pair similarities and merging all link pairs into a dendrogram, we can construct the inner structure of the multilayer networks. After that, by finding the maximum community density for this dendrogram, we can reveal the most suitable communities in these networks. The detailed steps of our method are described as follows:

**Step 1** Merging of Layers. Merging all layers into one merged graph can allow us to easily extract all layer pairs.
**Step 2** Link-Pair Abstraction. The link pair extracted from one merged graph should naturally contain a same-layer edge pair and a cross-layer edge pair.
**Step 3** Link-Pair Similarity Calculation. The similarity of a link pair is the combination of same-layer edge-pair similarity and cross-layer edge-pair similarity.
**Step 4** Hierarchical Structure Construction. By reordering all similarities in descending order and merging all these similarities in this order, we can construct the hierarchical structure of the multilayer networks.
**Step 5** Dendrogram Cutting and Potential Community Evaluation. Once the dendrogram has been formed, each height in this dendrogram represents a type of community.

**Fig 4:**
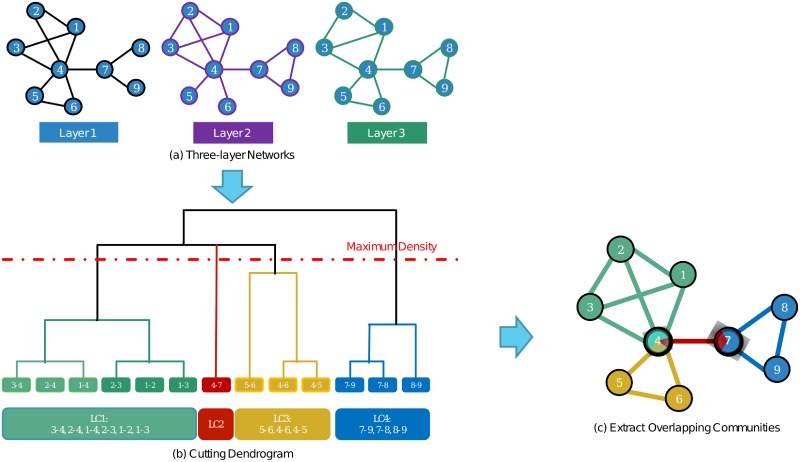
A toy example of how our algorithm works.

Here, we use Fig 4 to illustrate the whole algorithm. First, we use sub-graph (a) to represent a three-layer network. For sub-graph (b), it contains three steps: a) By calculating the similarities of link pairs, we can form a dendrogram. b) By calculating the community density for each height of the dendrogram, we can find the maximum density. c) By using this density, we can divide the dendrogram into four communities. Finally, for sub-graph (c), four overlapping communities can be easily extracted.

#### Time complexity analysis

Take a |*L*|-layer multilayer networks *MN* = (*V*, *E*) that has *M* link-pairs and *M*′ different similarities as an example. If the node sets are different from one layer to another, then we have to consider all the layers, which means that the node number that we must consider is $\left|V\right|=\sum_{l=1}^{L}V_{l}$. On the contrary, if the node sets remain the same throughout all layers, then the total number that we should consider is |*V*| = *V*_*l*_. Moreover, as each layer has its own topological structure, then the total edge number we should take into consideration is $\left|E\right|=\sum_{l=1}^{L}E_{l}$. After obtaining this pre-knowledge, we can analysis the time complexity for each step as follows:

**Step 1** : As the algorithm has to traverse all the nodes and edges through all layers, the time complexity is *O*(|*V*| + |*E*|).
**Step 2** : For each edge, there are two node neighbors that we should consider. The worst case is that this node connects to other nodes in the merged graph, which makes the worst time complexity as *O*(|*E*| × *log*_2_|*V*|).
**Step 3** : To calculate the link-pair similarity, we should not only look up all the graphs to find the same-layer and cross-layer behavior in it, but also must extract all the nodes’ neighbors from different layers. Moreover, the worst case is similar to step 2. The worst time complexity is *O*(|*L*| × *M* × *log*_3_|*V*|).
**Step 4** : If the layer number of the dendrogram is equal to the number of different link-pair similarities, then the worst time complexity is *O*(*M*′).
**Step 5** : To evaluate the community density, we need all layers to participate; in this case, the worst time complexity is *O*(*M*′ × |*L*|).

## Results

### Synthetic networks

The primary idea on how to create a graph with ground truth comes in two parts: 1). We use the Barabási-Albert model to simulate each subgraph topology, and 2) we apply a “random connection/disconnection adding strategy” to add/delete edges among subgraphs to construct a graph. Here, to predetermine communities within our synthetic network, we mark nodes within the same subgraph as the same community. To evaluate the “recall rate” and “precision rate” metrics for our method, we build two types of multilayer networks to assess each of these metrics.

#### Recall rate

**Fig 5:**
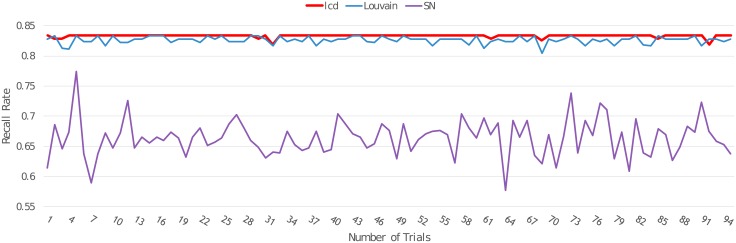
Recall rate comparison of our method and two other methods on synthetic multilayer networks.

For recall rate, as this metric focuses on how many nodes have been detected in a community, it mainly focuses on the nodes that can be successfully retrieved. On this basis, we create three-layer multilayer networks with three subgraphs, and each subgraph contains 10 nodes. Moreover, for each network, we randomly choose two nodes to join to other subgraphs as noises. The detailed information on how to create these simulated networks (Dataset A) are included in the Supporting Information (see Dataset A in S1 Appendix). The performance on the recall rate is shown in Fig 5. We applied our method (lcd), the Louvain method (Louvain), and link community detection (SN) for each three-layer multilayer network and calculated the recall rate (vertical axis) for each method. Moreover, we calculated the recall rate of these three methods for 100 synthetic three-layer multilayer networks (horizontal axis). In this figure, the red line stands for our method’s results, the blue line represents the Louvain method’s results, and the purple line represents the link community detection method’s results. From this validation, we can see that our method had the highest recall rate, whereas the link community detection method had the lowest.

#### Precision rate

**Fig 6:**
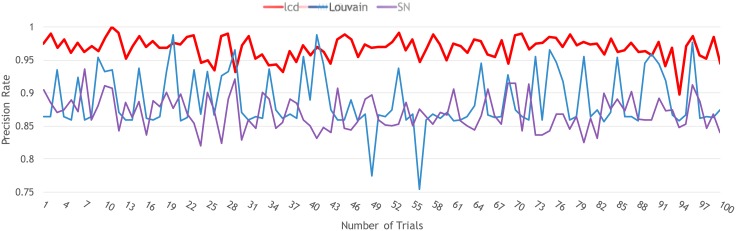
Precision rate comparison of our method and two other methods on synthetic multilayer networks.

For the precision rate, as this metric is the fraction of how many nodes have been divided into the right community, it is mainly focused on the robustness of the community discovery algorithms. Here, we created a multilayer network with three communities, each community contains 20 nodes. Moreover, we added 10 more nodes as noise nodes, so that they can randomly choose the connection behaviors with other nodes in each layer. The reason why we chose this type of synthetic network for the validation of the precision rate is that the precision rate can determine which algorithm is robust and accurate enough to reveal true communities. The detailed information on how to create this simulated network (Dataset B) is included in the Supporting Information (see Dataset B in S1 Appendix). The performance on the precision rate is shown in Fig 6. The horizontal axis shows the 100 synthetic three-layer multilayer networks, whereas the vertical axis shows the precision rate for our method (lcd), the Louvain method (Louvain), and the link community detection method (SN). Here, the red line stands for our method’s results, the blue line represents the Louvain method’s results, and the purple line represents the link community detection method’s results. From this validation, we can see that our method had the highest precision rate of around 97%, whereas the accuracy rates of the Louvain method and link community detection method were relatively low.

### Real-world networks

**Fig 7:**
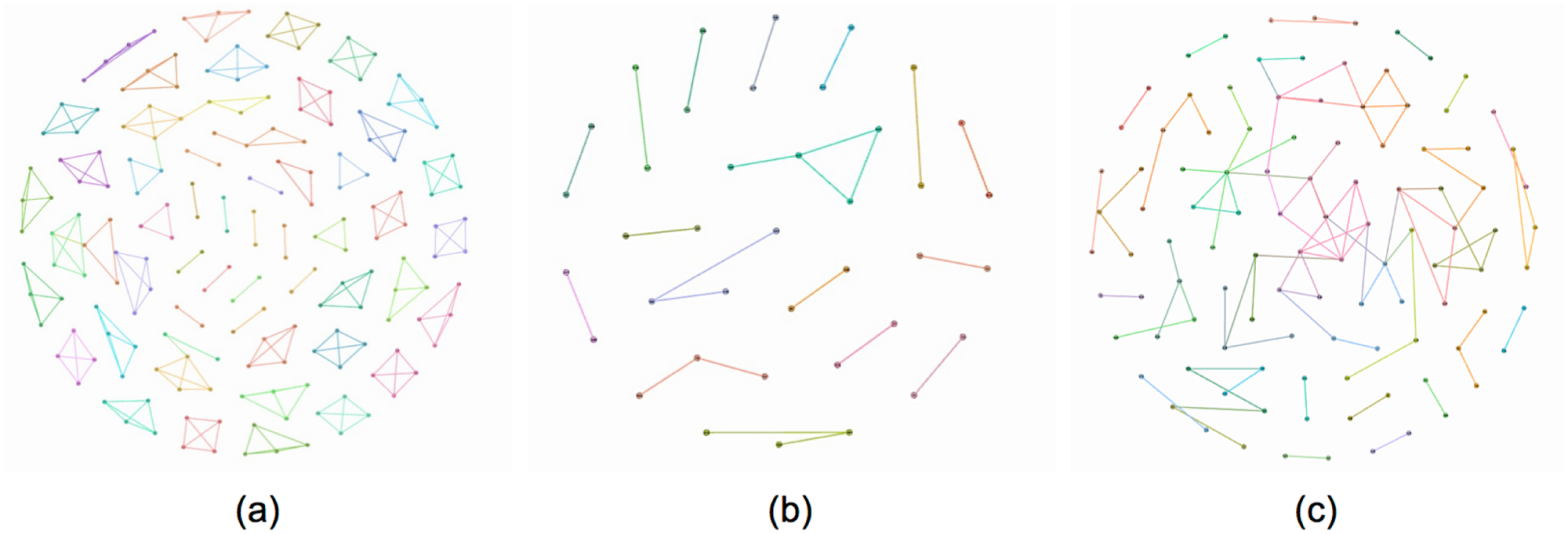
Three-layer multilayer networks on students’ cooperation behaviors. (a) Partner Network with 183 nodes and 240 edges. (b) Computer Network with 39 nodes and 23 edges. (c) Time Network with 106 nodes and 97 edges.

To further illustrate the effectiveness of our methodology, here, we introduce the “Students’ Cooperation Social Networks” built for the computer and network security course at Ben-Gurion University [40]. This course requires students to log in to a particular website to present their papers. By analyzing the logs on that website, we can get the partner’s links, computer links, and time links between students to form a three-layer multilayer network (see Fig 7). Here, 1) The “Partner’s Network” stands for the partnerships between students if they worked together to finish a paper. 2) The “Computer Network” represents the students who finished their papers on the same machine, which will reflect the partnerships between students to some degree. 3) The “Time Network” represents another type of connections; although two students might use different computers to do their paperwork, we can extract the partnerships between them on the basis of the time similarity when they submitted their papers. For example, if two students submitted their papers at the same time, it would also give us a reason to believe that these two students might also have cooperation relationships in co-working the papers. In conclusion, all these three-layer networks can reflect the obvious or hidden connection behaviors between students from three different aspects, and, therefore, co-analyzing these networks may allow us to discover the overlapping communities in these students.

**Fig 8:**
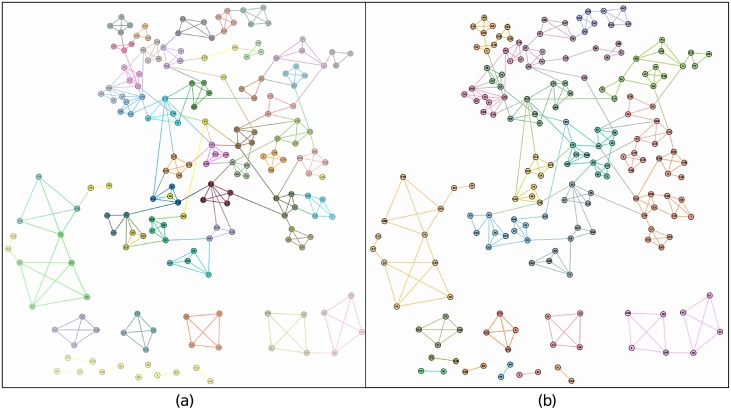
Comparison between our method and the Louvain method.

As the evaluation results from synthetic datasets showed that the link community discovery method was relatively not that good, and also as shown in the Fig C in S1 Appendix, we also used the partition results from this method to show that this method is not robust enough against noises contained in each layer. Hence, we only compared our method with the Louvain method on the three-layer multilayer networks. Fig 8 shows the community results. In this figure, the different colors in each subgraph express the various communities. As this dataset represents the partnership between students when they worked on a paper, it means that a community for students can be regarded as a small group of people who work in close cooperation to finish a paper. The ground truth information for partnership also shows that, for each partnership community, there were no more than four students (Please see http://proj.ise.bgu.ac.il/sns/students.html for more details) in it.

By applying our method and the Louvain method on these three-layer multilayer networks, and as the size of this dataset is small enough, we could directly give the outcomes for each method. Sub-figure (a) stands for the communities from our method, and sub-figure (b) represents the communities by applying the Louvain algorithm. We can clearly see that our method (sub-graph (a)) could detect relatively small communities more accurately, whereas the Louvain method just merged them together.

After demonstrating the community results for our method, we want to give a detailed analysis for the moderation parameter *α* for Eq 5. On the basis of the Student’s Cooperation Social Networks, we try to give a detailed information on how the partition results will respond according to the different values of *α*. In addition, we also illustrate how the partition results affect both same-layer and cross-layer similarity simultaneously. All experiment results indicated that, when the same-layer similarity and cross-layer similarity had the same weight, the partition results would be best.

#### How *α* affects the cross-layer similarity

Given a link pair (*L*_1_, *L*_2_) and a moderation parameter *α*, Eq 5 shows how to use *α* to weigh the cross-layer similarity. As *α* only affects the cross-layer similarity, we used this equation to analyze the importance of cross-layer similarity. To evaluate the outcome, we introduced a “non-trivial community number” and a “max partition density” as evaluation indexes. Here, non-trivial community number indicates that the community size was larger than three, whereas the max partition density was calculated through our community evaluation method discussed in the Methods section. By considering “non-trivial” communities, we could evaluate how many meaningful communities can be abstracted by our method.

**Fig 9:**
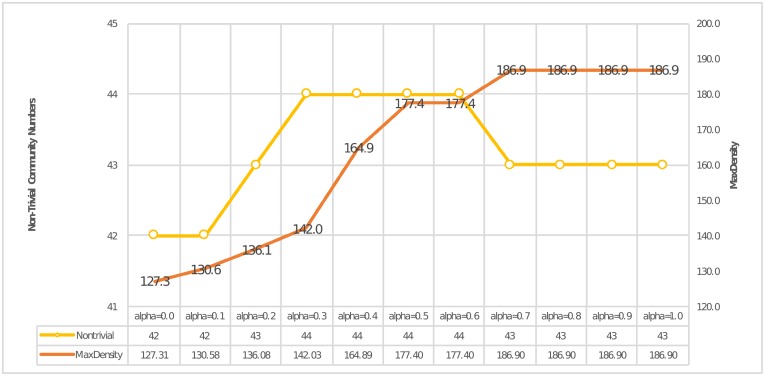
How *α* affected the cross-layer similarity.

Fig 9 shows the evaluation results. The left vertical axis stands for the community number of non-trivial communities, whereas the right vertical axis represents the density. From this figure, we can see that, when *α* > 0.6, the density became the largest one. Moreover, no matter how the moderation parameter changed, the number of nontrivial communities seemed to remain stable between {42, 44}. We think that the main reason why this phenomenon happens is that, despite the interplay between layers, the main behaviors of how two nodes are connected with each other are mainly preserved in each layer. However, here is another problem: Is same-layer similarity sufficient enough to depict the behaviors? Alternatively, do we really need cross-layer similarity?

**Fig 10:**
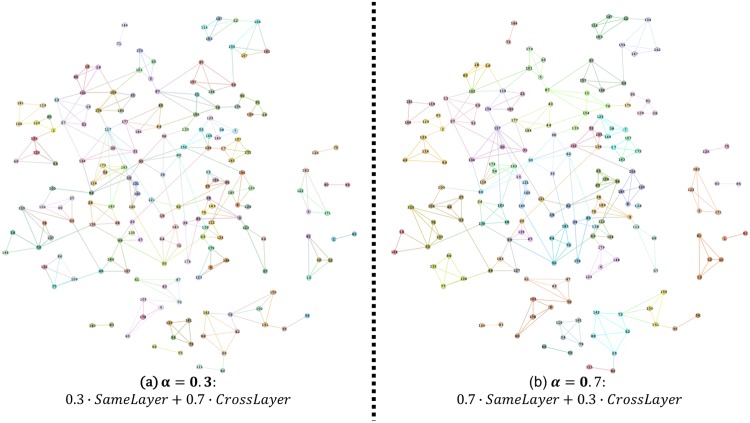
Comparison of different values of *α* on the cross layers.

To answer these questions, we use Fig 10 to demonstrate the partition results for *α* = 0 in subgraph (a) and *α* = 1 in subgraph (b). According to Eq 5, the former subgraph only considered the same-layer similarities, whereas the latter considered both the same-layer similarity and the cross-layer similarity. As for the Students’ Cooperation Social Networks, a logical community in these three-layer networks is a small group of students who co-operated with each other tightly. This feature indicates that this sub-group should be as dense as a clique. However, from this figure, we can tell that the communities in subgraph (a) were not dense enough. For example, take a look at the lower left corner of these two subgraphs, the node set {167, 162, 155, 104} should be in the same community, but for subgraph (a), this node set split into two sub-communities. In addition, the node set {17, 183, 107, 172} in the upper right corner should also be a community. From these comparisons, we can easily determine that using only same-layer similarity is not sufficient enough to find all the communities accurately in multilayer networks. This means that, in general, the cross-layer similarity is also a crucial and non-ignorable feature for detecting the right communities in multilayer networks.

#### How *α* affects both similarities

Given a link pair (*L*_1_, *L*_2_) and a moderation parameter *α* ∈ [0, 1], Eq 7 shows how to use *α* to weigh both similarities. As *α* can affect same-layer similarity and cross-layer similarity simultaneously, it is easy to notice that the higher the *α* is, the greater the influence of same-layer similarity is on link-pair similarity, and vice versa.

$$\begin{array}{c} SIM\left(L_{src↔mid},L_{mid↔dst}\right)=\alpha \times samelayerSim\left(L_{src↔mid},L_{mid↔dst}\right)\\ +\left(1-\alpha \right)\times crosslayerSim\left(L_{src↔mid},L_{mid↔dst}\right) \end{array}$$ (7)

**Fig 11:**
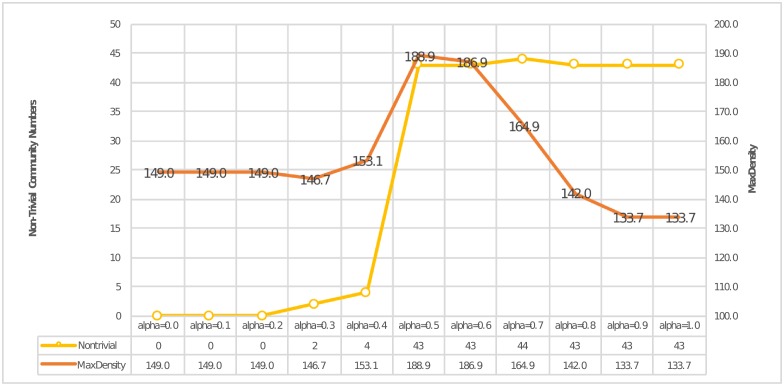
How *α* affected both similarities.

By applying the moderation parameter *α* from 0 to 1 in the link-pair similarity calculation, we can demonstrate from the experimental results how the partition results can be affected by the different proportion of same-layer and cross-layer similarities. Fig 11 shows the evaluation results. In this figure, we also used the non-trivial community number and max partition density as evaluation indexes. From this figure, we can see that there was a peak in *α* ∈ [0.5, 0.6] for both indexes and that after *α* > 0.4, the number of “non-trivial” communities became reasonable. This figure gives us a concrete idea that we should take not only the “same-layer” link pair but also the “cross-layer” link pair into consideration. Ignoring any of them will lead to a misunderstanding.

**Fig 12:**
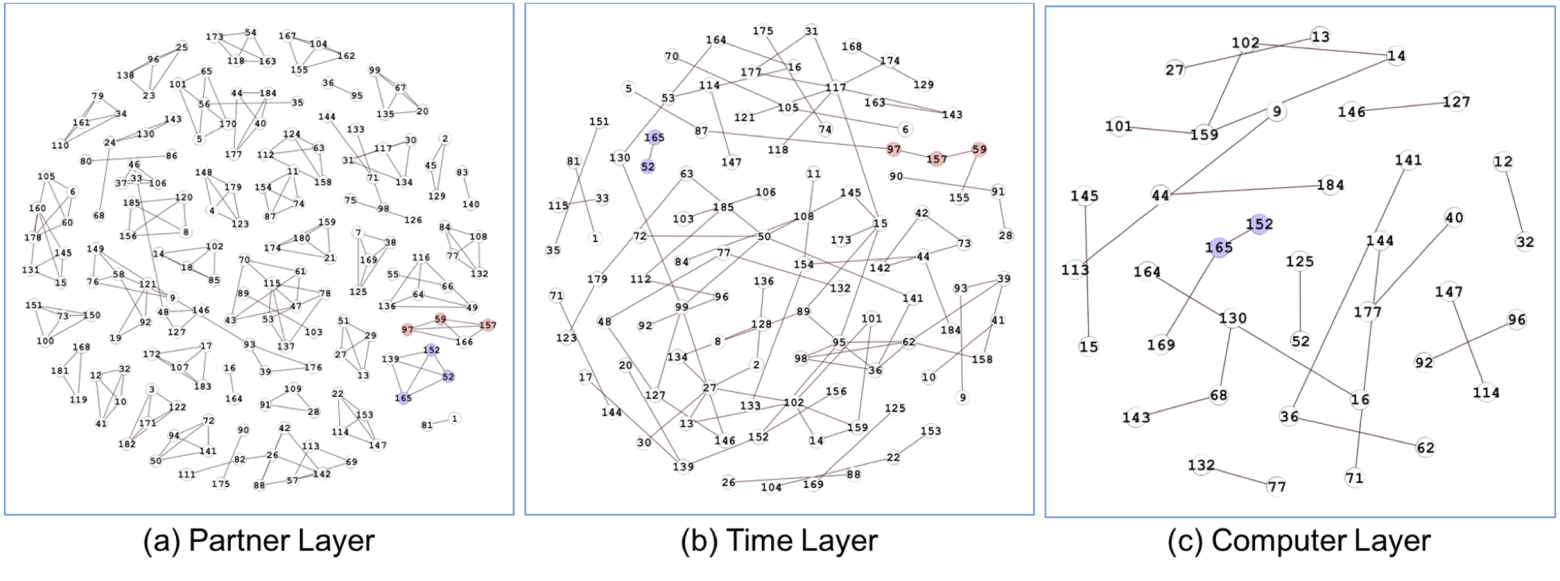
Node behaviors in the original three-layer networks.

**Fig 13:**
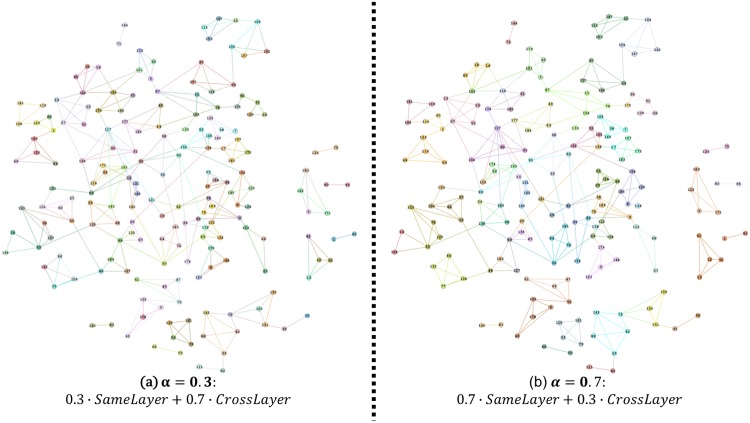
Comparison of different values of *α* on both layers.

Here, we give a detailed analysis for each step of *α*:

- 0 ≤ *α* ≤ 0.2**:** There exist no “non-trivial” communities. This phenomenon means that, although cross-layer edge pairs do a good job of preserving the important interplays, we cannot use this piece of information without taking same-layer edge pairs into consideration. After all, node behavior should exhibit same-layer similarity first.
- 0.3 ≤ *α* ≤ 0.4**:** By increasing *α*, our method begins to consider the structural topology information in each layer. Take Fig 12 as an example, our method detected two communities: {59, 97, 157} and {52, 152, 165}. Fig 12 shows the original connecting behaviors for these nodes in three-layer networks, where the red nodes represent the former community and the purple nodes represent latter one. This figure shows that edge (52, 165) existed in all layers and that edges (52, 165), (59, 157) and (97, 157) existed in two layers. Thus, it is natural to divide these nodes into communities.
- 0.5 ≤ *α* ≤ 0.6**:** Different from previous partition results, the number of non-trivial communities and max density rose sharply from this step, and the density achieved the global optimal value in *α*. Moreover, the number of non-trivial communities became logical. In our opinion, we believe that this phenomenon provides strong evidence that same-layer similarity must be considered.
- 0.7 ≤ *α* ≤ 1**:** With the further increase of the parameter *α*, the number of non-trivial communities tended to remain stable, and numerous communities had already been revealed by our method. Take Fig 13 as an example; here, we compared *α* = 0.3 (subfigure (a)) and *α* = 0.7 (subfigure (b)). From the comparison, we can easily see that numerous dense and small communities had been revealed in subfigure(b). However, the value of max density decreased. We think that this phenomenon shares the same reason as what we discussed in Fig 10; that is, without considering the cross-layer similarity, our method would be vulnerable to the noises in same-layer structural topologies.

Therefore, in general, a parameter *α* that is either too high or too low will affect the partition results. At the same time, we should not ignore the effectiveness of cross-layer edge-pair similarities. Therefore, we suggest that same-layer similarity and cross-layer similarity should be considered simultaneously and equally.

## Discussion

### Advantages of our method

As a departure from merging networks or communities, our method do leverage both in and cross layers’ topology information. Moreover, unlike any other overlapping community discovery methods in multilayer networks or multiplex networks, our method treats edge pairs in layers and cross layers as the fundamental elements to form communities. In general, we mainly focus on how to capture the interplay between layers. By borrowing the idea from Rosvall et al. [16, 41–44] to presume that, we can regard “cross-layer edge pair” as a “bridge” that a random walker can use to switch layers. Moreover, the similarity for two cross-layer edges stands for how many random walkers will switch layers by going through this “bridge.” Unlike multi-slice modularity methods, which mainly focus on the tightness of a subset of nodes in layers and cross layers, or Infomap-related methods, which only pay attention to a module that captures the information flow for a relative long time, our method combines the advantage of these two types of popular community detection methods by regarding both “same-layer” and “cross-layer” edge pairs as a way for information to flow, and we propose a way to calculate the tightness of the community. In addition, after forming the hierarchical structure for the multilayer networks, we designed a novel metric that can evaluate the community density in multilayer networks, rather than use a simple upgrade from community density in a single network.

### Weakness of our method

**Fig 14:**
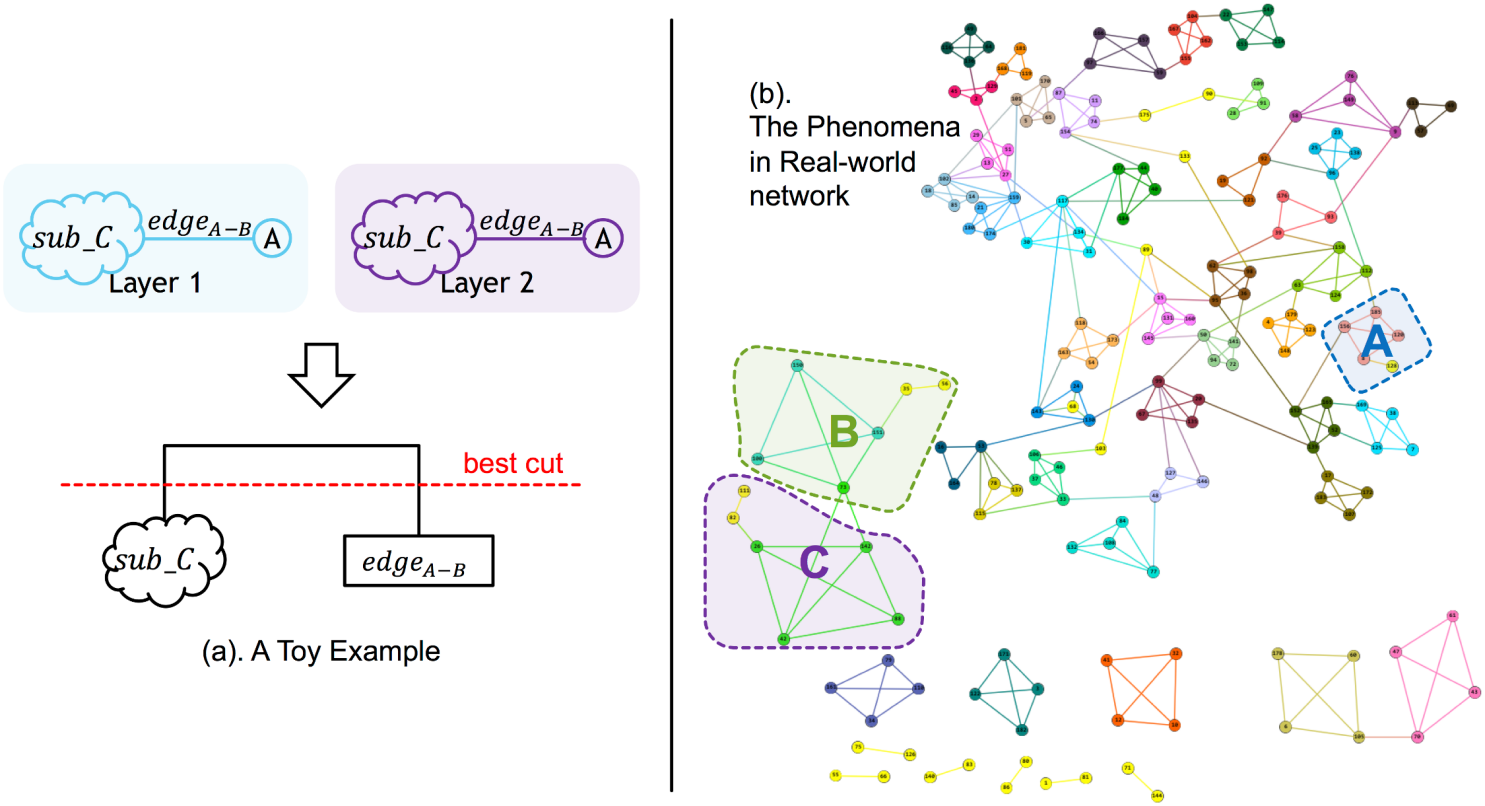
Weakness of our method.

We have to say that, although the evaluation results from both synthetic datasets and real-world dataset showed that our method can divide most edges into right communities, there is an instance wherein our method may fail: if two parts of a community are merged above the “cutting criterion,” our method may consider treating these two parts as two separate communities. Take Fig 14 as an example. Sub-figure (a) represents a toy example. For a two-layer network, if there is a sub-community *Sub*−*C* and an *edge*_*A*−*B*_, we can form a dendrogram. The red line stands for the best community density. By following this red line, we can get two communities: one is the sub-community *Sub*_*C* and the other is *edge*_*A*−*B*_. Actually, however, the right community should contain these two parts together. Sub-figure (b) represents the mispartitioned part for the real-world network. Here, for each community in the blue part (*A*), green part (*B*), and purple part *C*, these nodes should be merged into one community instead of two. We believe that there may be two ways to solve this weakness: 1) To calculate the neighbors of a small community, if the number is quite small, then we can directly merge this small community into a big community beside it. 2) The cutting criterion should become adaptive, which means that this criterion is no longer a straight line, but can move up and down with the height of the dendrogram. These suggestions may allow us to design a more accurate and reasonable community discovery method, which we will leave for future work.

Generally speaking, despite some weaknesses, by leveraging the topology information in each layer and the interplay between layers, our method can reveal the overlapping communities in multilayer networks. Moreover, we believe that our method can become a useful tool for practical applications in social network analysis. In addition, as shown in a series of recent publications [45, 46], large-scale graph processing and incremental graph analysis on dynamic data streams have become more and more vital in social network analysis.

## Conclusion

In this paper, we introduced a novel method for finding overlapping communities in multilayer networks. Our method has the ability to preserve the topology information within each layer and leverage the interplay between layers. By taking both same-layer and cross-layer edge pairs into consideration, our method forms a hierarchical structure (dendrogram) of the multilayer networks, where the fundamental elements in this dendrogram are the edges. Then, by introducing a new community density metric for multilayer networks, we cut this dendrogram to generate overlapping communities according to community density maximization. For evaluations on synthetic networks specially designed for “recall rate” and “precision rate,” our method can uncover true communities without being affected by the noises in the layers. For real-world networks, our method can find smaller and denser overlapping communities. Moreover, we also used a real-world dataset to prove that same-layer and cross-layer edge pairs should all be taken into consideration.

In our future work, we will test our method on more real-world datasets and apply our method to various types of applications in social network analysis, such as “recommendation systems” [47–49], “socially aware computing” [50, 51], etc.

## Supporting information

S1 Appendix Synthetic datasets introductions and results. (PDF)
