# Supplementary material for: Finding overlapping communities in multilayer networks
Source: PLoS One. 2018 Apr 25;13(4):e0188747. doi: 10.1371/journal.pone.0188747 (PMC5919045; doi:10.1371/journal.pone.0188747)
Supplement: S1 Appendix — (PDF) [file pone.0188747.s001.pdf]

Finding Overlapping Communities in Multilayer Networks

Weiyi Liu[1,2], Toyotaro Suzumura[2], Hongyu Ji[1], Guangmin Hu[1*],

1 University of Electronic Science and Technology of China, School of Communication & Information Engineering, Chengdu, Si Chuan, China
2 IBM Thomas J. Watson Research Center, Network Science and Big Data Analytics Department, New York, U.S.A

* hgm@uestc.edu.cn

Supporting information

Dataset A: Random access in other communities in each layer. Here, we introduce how to generate Dataset A in detail. Figure.A gives an example of how this dataset looks like. For each node, we give the unique name for each node by combining the community ID and the node ID together. For example, a node GX_Y indicates that node Y is in community GX. In addition, we use the colors purple, yellow, and green to represent community $G0$, $G1$, and $G2$, respectively.

Subfigure (a) represents a layer of this synthetic multilayer network. We use light colors to represent nodes that join other communities in this layer. In detail: node $G2\_0$ and $G1\_3$ join community $G0$, node $G2\_9$ joins community $G1$, and nodes $G0\_2$, $G0\_6$, and $G1\_0$ join community $G2$.

Subfigure (b) illustrates the results of our method and the "multi-slice modularity"-based Louvain method. The color of the nodes stands for the partition results for the Louvain method, and the color of the edges stands for the edge partition for our method. Please note that, although we use edges to form a dendrogram, the outcome of our method should be node-based communities, which means the edge color here is just a middle step instead of the final output. In addition, because we reported in the Discussion section that our method may generate some two-node communities, these "trivial communities" will not affect our result; therefore, we simply hide the edges of these small-enough communities (most of them are two-node communities) to make the result much clearer to see. From this subgraph, we can tell that the Louvain method incorrectly assigned node $G1\_5$ to community $G0$ and node $G2\_4$ to community $G1$. For our method, all the nodes were assigned to the right communities, and we also find overlapping nodes such as $G0\_1$, $G0\_6$, $G1\_5$, and $G2\_4$.

Dataset B: Adding noises to each layer. Here, we introduce how to generate Dataset B in detail. Take Figure.B as an example. The notation for the nodes in Figure.B is the same as with Figure.A. Moreover, we used the colors purple, red, and green to demonstrate community $G0$, $G1$, and $G2$, respectively. Moreover, we also used label N to denote the noise nodes. Subfigure (a) demonstrates a layer with 60 nodes and 10 noise nodes, and a layer is composed of three communities from these nodes, and then we randomly inject all noise nodes into these communities and let them choose their neighbors randomly. Take subfigure (a) as an example, we used red/blue/green circles to illustrate the three noise nodes: N_7, N_5, and N_0. In addition, to increase the influence of random noises for the layers, we increased the number of random connections between random noise nodes and the communities in each layer. It can be seen clearly that subfigures (b) and (c) have more connections in a community and also

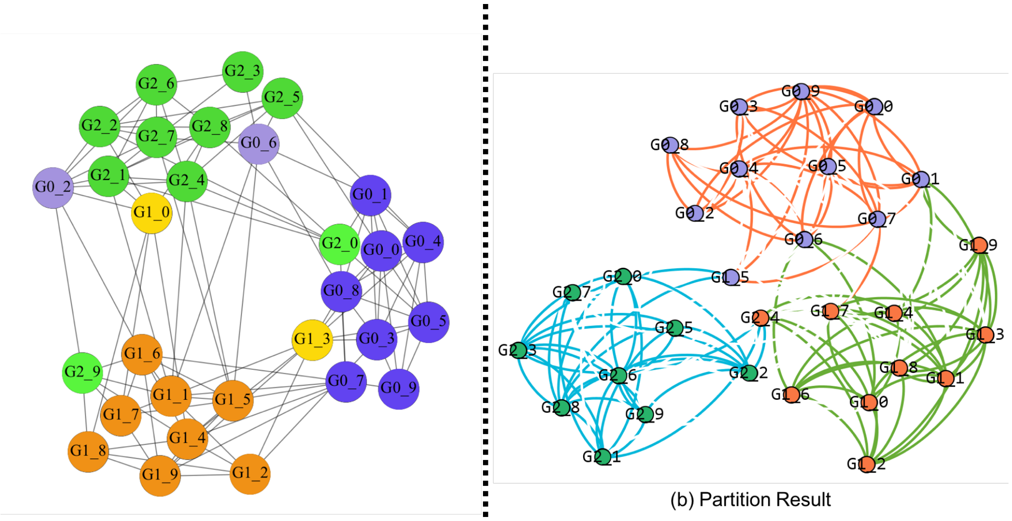

(a) One Layer Example (b) Partition Result

Figure A. Toy Example and Partition Results for Dataset A.

between communities than subfigure (a). Moreover, in subfigure (c), with the use of the Louvain method on this graph, there even emerged a new yellow community N_8, N_4, N_0.

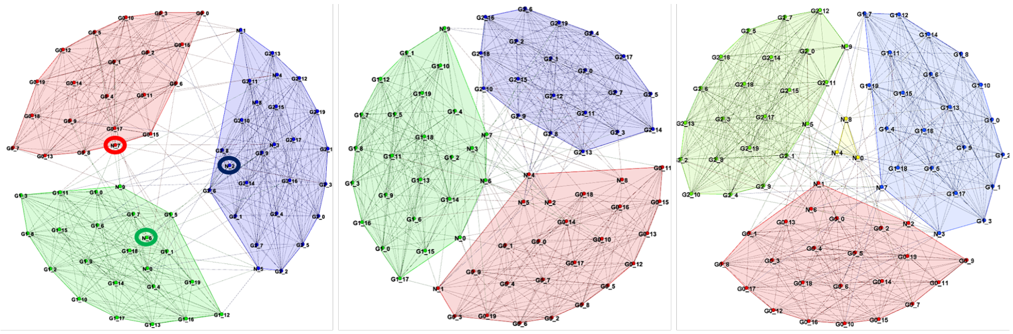

(a) Layer One (b) Layer Two (c) Layer Three

Figure B. A three-layer multiplex network created from Synthetic Dataset B.

Figure.C shows the partition results: subfigure (a) represents our methods and the multi-slice modularity-based Louvain method; subfigure (b) illustrates the results of the link community discovery method in a unified graph. Moreover, we also used node colors to represent communities from the Louvain method and used edge colors as a middle outcome for our method and the link community discovery method.

For the Louvain results in subfigure (a), we can tell that all 60 nodes were assigned to the right communities. However, all noise nodes were also assigned to different communities. Here, the noise node set N_1,N_2,N_3,N_5,N_8,N_9 was assigned to community $G0$ and N_0,N_4,N_7 were divided into community $G2$. We can see that our method not only divided 60 nodes into right communities, but also could distinguish all noise nodes without assigning them to any of the communities. In our opinion, we think that it is logical that all these noise nodes should not be in a community or in several communities as they are random noises. Please note that, for this particular example, by using different edge colors to represent different communities, we also found overlapping nodes in community $G0$. Actually, this community is composed of four

types of sub-communities: 1) a dark color related edge community, which has 62 edges; 2) a yellow color related edge community, which has 16 edges; 3) a red color related edge community, which has 5 edges; and 4) a pink color related edge community, which has 5 edges. See Table: Edge details for detailed information.

For the link community discovery method, in the weighted graph shown in subfigure (b), it is easy to draw the conclusion that this method cannot detect right communities, nor can it resist noises.

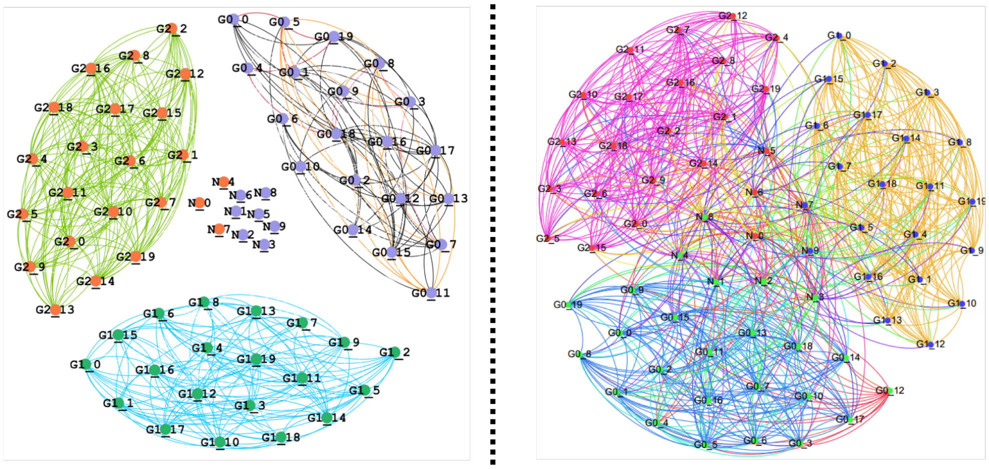

(a) Partition Results for Our method and Louvain method

(b) Partition Results for Link Community Discovery method

Figure C. Partition Results for Synthetic Dataset B.

Table. Edge details.

Color: DARK

Source	Target	Source	Target	Source	Target
G0_7	G0_2	G0_19	G0_15	G0_12	G0_15
G0_7	G0_10	G0_19	G0_0	G0_12	G0_8
G0_7	G0_12	G0_19	G0_16		
G0_7	G0_15	G0_19	G0_3		
G0_7	G0_6	G0_19	G0_17		
G0_7	G0_16	G0_4	G0_2		
G0_7	G0_17	G0_3	G0_0		
G0_10	G0_15	G0_3	G0_17		
G0_10	G0_5	G0_3	G0_14		
G0_10	G0_17	G0_17	G0_2		
G0_10	G0_8	G0_17	G0_9		
G0_18	G0_15	G0_17	G0_12		
G0_18	G0_11	G0_17	G0_15		
G0_18	G0_1	G0_17	G0_0		
G0_18	G0_16	G0_17	G0_16		
G0_18	G0_17	G0_17	G0_13		
G0_18	G0_2	G0_17	G0_14		
G0_18	G0_12	G0_11	G0_13		
G0_18	G0_19	G0_11	G0_6		
G0_18	G0_0	G0_13	G0_12		
G0_18	G0_13	G0_13	G0_2		
G0_18	G0_14	G0_13	G0_15		
G0_1	G0_2	G0_13	G0_16		
G0_1	G0_17	G0_16	G0_15		
G0_1	G0_15	G0_16	G0_0		
G0_1	G0_0	G0_2	G0_15		
G0_1	G0_19	G0_2	G0_12		
G0_1	G0_16	G0_2	G0_6		
G0_1	G0_3	G0_0	G0_15		
G0_1	G0_14	G0_12	G0_6		

Color: YELLOW

Source	Target
G0_7	G0_8
G0_7	G0_5
G0_18	G0_5
G0_19	G0_11
G0_3	G0_5
G0_17	G0_11
G0_11	G0_2
G0_11	G0_15
G0_11	G0_16
G0_11	G0_5
G0_11	G0_8
G0_13	G0_5
G0_13	G0_8
G0_2	G0_5
G0_9	G0_8
G0_5	G0_8

Color: RED		Color: PINK	
Source	Target	Source	Target
G0_18	G0_4	G0_7	G0_3
G0_1	G0_5	G0_18	G0_9
G0_19	G0_4	G0_18	G0_8
G0_19	G0_5	G0_3	G0_9
G0_0	G0_5	G0_3	G0_8
